# Medical School Admissions After the Supreme Court’s 2023 Affirmative Action Ruling

**DOI:** 10.1001/jamanetworkopen.2025.27008

**Published:** 2025-08-26

**Authors:** Mytien Nguyen, Alexandra M. Hajduk, Tonya L. Fancher, Mark C. Henderson, Jeph Herrin, David Henderson, Judee Richardson, Shruthi Venkataraman, Laura Castillo-Page, Soo-Min Shin, Meghan O’Connell, Adeola Ayedun, Dowin Boatright, Sarwat I. Chaudhry

**Affiliations:** 1 Department of Immunobiology, Yale School of Medicine, New Haven, Connecticut; 2 Section of Geriatrics, Department of Internal Medicine, Yale School of Medicine, New Haven, Connecticut; 3 Division of General Internal Medicine, University of California, Davis, School of Medicine, Sacramento; 4 Department of Internal Medicine, University of California Davis School of Medicine, Sacramento; 5 Office of Medical Education, University of California Davis School of Medicine, Sacramento; 6 Section of Cardiology, Department of Internal Medicine, Yale School of Medicine, New Haven, Connecticut; 7 American Medical Association, Chicago, Illinois; 8 Department of Emergency Medicine, New York University Grossman School of Medicine, New York, New York; 9 National Academies of Science, Engineering, and Medicine, Washington, District of Columbia; 10 Now with American Board of Medical Specialties, Chicago, Illinois; 11 Section of General Internal Medicine, Department of Medicine, Yale School of Medicine, New Haven, Connecticut

## Abstract

**Importance:**

The 2023 Supreme Court of the United States (SCOTUS) *Students for Fair Admissions, Inc. v President and Fellows of Harvard College* ruling to restrict race-based affirmative action is a landmark decision with potentially far-reaching consequences.

**Objective:**

To examine application, acceptance, and matriculation rates into doctor of medicine (MD)–granting programs before and after the 2023 SCOTUS decision.

**Design, Setting, and Participants:**

This retrospective cross-sectional study used deidentified data from the Association of American Medical Colleges on applicants and matriculants to US MD-granting medical schools 5 years before (2019-2023) and 1 year after (2024) the SCOTUS ruling.

**Main Outcomes and Measures:**

Application, acceptance, and matriculation into MD-granting programs.

**Results:**

The study sample included 291 764 applicants to MD programs between 2019 and 2024 (162 211 identifying as female [55.59%]; 4576 American Indian, Alaska Native, Native Hawaiian, or Pacific Islander [1.57%], 84 122 Asian [28.83%], 34 256 Black [11.74%], 35 707 Hispanic [12.24%], and 154 621 White [53.00%]). Compared with mean acceptance rates in 2019 to 2023, acceptance rates in 2024 were higher for White (40.37% vs 47.47%; difference, 7.10 percentage points [95% CI, 6.21 to 7.98 percentage points]; *P* < .001) and Asian (38.26% vs 45.19%; difference, 6.93 percentage points [95% CI, 5.78 to 8.07 percentage points]; *P* < .001) applicants and lower for Black applicants (36.06% vs 33.08%; difference, −2.98 percentage points [95% CI, −4.74 to −1.21 percentage points]; *P* < .001) and underrepresented in medicine (URiM) applicants overall (39.68% vs 38.33%; difference, −1.35 percentage points [95% CI, −2.60 to −0.09 percentage points]; *P* = .02). No racial or ethnic difference in acceptance rates were observed in 2019 to 2023, but in 2024, URiM applicants had significantly lower acceptance rates than White (difference, −9.14 percentage points; *P* < .001) and Asian (difference, −6.86 percentage points; *P* < .001) applicants. Comparing the mean of 18 304 matriculants in 2019 to 2023 with 19 018 matriculants in 2024, White student representation decreased from 10 132 matriculants (55.35%) to 10 158 matriculants (53.41%) for a decrease of 1.94 percentage points (95% CI, −3.31 to −0.56 percentage points; *P* = .009), Asian student representation increased from 5102 matriculants (27.87%) to 6288 matriculants (33.06%) for an increase of by 5.19 percentages points (95% CI, 3.49 to 6.88 percentage points; *P* < .001), and URiM student representation decreased from 4466 matriculants (24.39%) to 3963 matriculants (20.83%) for a decrease of 3.56 percentage points (95% CI, −5.34 to −1.77 percentage points; *P* < .001), equating to 503 fewer URiM matriculants in 2024. Post–SCOTUS decision declines in URiM representation were concentrated in schools located in states without prior state-level affirmative action bans (mean [SD] change, −6.14 [8.93] percentage points vs 0.10 [8.11] percentage points; *P* < .001).

**Conclusions and Relevance:**

In this study, URiM student matriculation into US medical schools declined after the 2023 SCOTUS ruling, with an emergent disparity in acceptance rates of URiM applicants relative to Asian and White students. The decline in URiM student matriculation was concentrated in states without a preexisting state-level affirmative action ban, suggesting that there may be an association between the SCOTUS ruling and demographic changes in matriculation.

## Key Points

**Question:**

What are the rates of acceptance and matriculation into doctor of medicine (MD) programs before and after the 2023 US Supreme Court decision on affirmative action?

**Findings:**

In this cross-sectional study of 291 764 applicants to MD programs, there was a significant decrease in acceptance rates for applicants from race and ethnicity groups who are underrepresented in medicine (URiM) in 2024 relative to 2019 to 2023, with an associated decline of 503 URiM matriculants to MD programs.

**Meaning:**

This study found that the percentage of URiM matriculants to MD programs declined after the 2023 Supreme Court ruling.

## Teaser

This cross-sectional study investigates rates of application, acceptance, and matriculation by race and ethnicity into doctor of medicine programs before and after the Supreme Court’s 2023 *Students for Fair Admissions, Inc. v President and Fellows of Harvard College* affirmative action decision.

## Introduction

Race-based affirmative action has been associated with gains in the racial and ethnic diversity of many professions, including medicine.^1,2^ On June 29, 2023, the Supreme Court of the United States (SCOTUS) struck down the use of the “educational benefits of diversity” as rationale for affirmative action in *Students for Fair Admissions, Inc. v President and Fellows of Harvard College*, effectively ending the use of race-conscious admissions in higher education.^3,4,5^

Previous, state-level affirmative action bans in 8 states (implemented 1997-2013) were associated with a 4.8% absolute decline in representation of students who identify as underrepresented in medicine (URiM) enrolled in public medical schools after 5 years.^6^ In January 2025, the Association of American Medical Colleges (AAMC) reported a decline in number of URiM matriculants to medical schools in 2024 compared with 2023.^7^ Building on this observation, our study aimed to provide a comprehensive, empirical assessment of the racial and ethnic characteristics of US medical school applicants, accepted students, and matriculants before and after the SCOTUS ruling. Furthermore, we examined how changes in racial and ethnic representation were associated with medical school characteristics that may influence the selection of students for medical school admission.

## Methods

This cross-sectional study was conducted according to the Strengthening the Reporting of Observational Studies in Epidemiology (STROBE) reporting guideline. The study was approved by the New York University Langone Health and Yale School of Medicine institutional review boards. Applicants provided electronic consent on the American Medical College Application Service (AMCAS).

### Data and Participants

We conducted a retrospective study of MD programs at US medical schools in academic years 2019 to 2024 using student-level data from the AAMC data warehouse. We limited our study to MD programs, excluding accelerated MD programs, combined BS-MD or BA-MD programs, and other combined programs, such as MD-MPH and MD-PhD programs, given that these programs have different admissions processes. To enhance the stability of estimates, we used the period 2019 to 2023 as the *pre–SCOTUS ruling period*, with 2024 serving as the *post–SCOTUS ruling period*. Although the SCOTUS decision was issued in June 2023, the admissions cycle for the 2023 entering class concluded prior to the ruling, and thus 2023 reflects admissions outcomes under preruling policies. Due to the increased number of applicants in 2021 and 2022, sensitivity analyses were performed using only 2023 as the pre–SCOTUS ruling period (eTables 2-5 in Supplement 1).

Race and ethnicity were self-reported by students in the AMCAS and examined for 6 non–mutually exclusive groups: American Indian, Alaska Native, Native Hawaiian, and Pacific Islander); Asian; Black; Hispanic; White; and other. Students who selected Hispanic were categorized as Hispanic regardless of racial identification. Students selected the *other* category on their AMCAS. URiM students were those who identified as American Indian, Alaska Native, Native Hawaiian, and Pacific Islander; Black; or Hispanic alone or in combination with other racial and ethnic identities.^8,9,10^ Students with missing race and ethnicity data were excluded from analyses.

### Outcomes

Applicants were defined as students who submitted 1 or more AMCAS application to an MD program, including first-time and repeat applicants. Acceptance into an MD program was defined as receiving an acceptance offer to 1 or more MD programs as reported by AMCAS.

### Medical School Characteristics

Medical school characteristics were provided by the AAMC and included state policies, ownership, geographic region, and mean Medical College Admissions Test (MCAT) score of matriculants in academic years 2019 to 2023. Due to the potential impact of statewide policies, schools were categorized by whether they were located in 1 of 9 states with state-level affirmative action bans in effect prior to 2023.^6^ Schools were also categorized based on whether they were located in 1 of 32 states with existing or proposed anti–diversity, equity, and inclusion (DEI) legislation as of June 2023.^11^

### Statistical Analysis

For our main analyses, we evaluated changes in proportions of applicants, acceptance rates, and proportions of matriculants to MD programs at the national and school level by race and ethnicity in the pre–SCOTUS (2019-2023) and post–SCOTUS (2024) ruling application cycles. To calculate the proportional representation of each race and ethnicity group among pre–SCOTUS ruling applicants, we first calculated the mean number of applicants in application cycles 2019, 2020, 2021, 2022, and 2023 overall and for each race and ethnicity group. We then calculated the proportion of applicants from each race and ethnicity group by dividing the mean number of applicants in a race and ethnicity group by the mean total number of applicants. We evaluated differences in proportions of applicants from each race and ethnicity group for 2019 to 2023 vs 2024 using χ^2^ tests. We calculated the acceptance rate for each race and ethnicity group in the pre–SCOTUS ruling period by first finding the mean number of acceptances for each group in 2019, 2020, 2021, 2022, and 2023 and dividing the mean number of acceptances by the mean number of applicants in that race and ethnicity group during the pre–SCOTUS ruling period. We evaluated differences in acceptance rates between the pre– and post–SCOTUS ruling periods overall and for each race and ethnicity group using χ^2^ tests. To calculate the proportional representation of each race and ethnicity group among pre–SCOTUS ruling matriculants, we first found the mean number of matriculants in application cycles 2019, 2020, 2021, 2022, and 2023 overall and for each race and ethnicity group, then calculated the proportion of matriculants from each race and ethnicity group by dividing the mean number of matriculants in a race and ethnicity group by the mean total number of matriculants. We then evaluated differences in proportional representation of each race and ethnicity group among matriculants for 2019 to 2023 vs 2024 using χ^2^ tests. We also conducted pairwise comparisons of acceptance rates between URiM and White applicants and URiM and Asian applicants in the pre– and post–SCOTUS ruling periods using χ^2^ tests. We performed sensitivity analyses to evaluate differences in acceptance rates and proportional representation of race and ethnicity groups among applicants and matriculants using 2023 data to define the pre–SCOTUS ruling period as an alternative to 2019 to 2023.

We conducted analyses at the school level to evaluate differences in the proportion of URiM matriculants at each school in the pre– and post–SCOTUS ruling periods according to school-level characteristics, including the presence of state policies (state anti-DEI policy and prior state-level affirmative action ban), ownership, geographic region, and mean MCAT score quartile. We evaluated differences in the mean school-level percentage of URiM matriculants for 2019 to 2023 vs 2024 using the Mann-Whitney *U* test. We evaluated differences in mean school-level percentages of URiM matriculants by school-level characteristic using Mann-Whitney *U* and Kruskal-Wallis tests, as applicable. We conducted sensitivity analyses using only 2023 data to represent the pre–SCOTUS ruling period.

All statistical analyses were performed (M.N. and A.M.H.) using Stata statistical software version 18.0 (StataCorp LLC). Statistical significance was determined using 2-sided *P* values < .05.

## Results

### Derivation of the Analytic Sample

After excluding 14 039 students with missing data on race or ethnicity, the main analytic sample included 291 764 applicants (95.41%; 162 211 female [55.59%]; 4576 American Indian, Alaska Native, Native Hawaiian, or Pacific Islander [1.57%], 84 122 Asian [28.83%], 34 256 Black [11.74%], 35 707 Hispanic [12.24%], and 154 621 White [53.00%]) who applied to at least 1 MD program in the application cycles for admission in 2019, 2020, 2021, 2022, 2023, or 2024. Applicant data were not available for 6 schools in the 2019 to 2023 period and for 2 schools in the 2023 to 2024 period; these schools were excluded from those school-level analyses.

### Racial and Ethnic Characteristics Before and After 2023 SCOTUS Ruling

#### Number and Percentage of Applicants

As shown in Table 1, there was a mean of 49 298 applicants per year to all MD programs for the period 2019 to 2023 compared with 45 275 applicants in 2024. The decline in number of applicants was seen for Black, Hispanic, White, and URiM students. There was an increase in Asian applicants and applicants with other race and ethnicity. Overall, there were 782 fewer URiM applicants in 2024 compared with 2019 to 2023 (Table 1).

**Table 1.** Race and Ethnicity of Applicants to Doctor of Medicine Programs

Race and ethnicity^a^	Applicants, No. (%)						
	2019	2020	2021	2022	2023	2019-2023 mean^b^	2024
All	46 031 (100)	47 134 (100)	56 279 (100)	49 767 (100)	47 278 (100)	49 298	45 275 (100)
American Indian, Alaska Native, Native Hawaiian, and Pacific Islander	759 (1.64)	748 (1.58)	891 (1.58)	731 (1.46)	772 (1.63)	780.2 (1.58)	675 (1.49)
Asian	12 274 (26.66)	12 895 (27.35)	15 551 (27.63)	14 573 (29.28)	14 362 (30.37)	13 931 (28.25)	14 467 (31.95)
Black	5050 (10.97)	5168 (10.96)	7310 (12.98)	5795 (11.64)	5500 (11.63)	5764.6 (11.69)	5433 (12.00)
Hispanic	5342 (11.6)	5654 (11.99)	7122 (12.65)	6049 (12.15)	5885 (12.44)	6010.4 (12.19)	5655 (12.49)
White	25 635 (55.69)	25 893 (54.93)	29 548 (52.5)	26 399 (53.04)	24 589 (52)	26 412.8 (53.57)	22 557 (49.82)
Other^c^	2202 (4.78)	2342 (4.96)	2848 (5.06)	2518 (5.05)	2393 (5.06)	2460.6 (4.99)	2515 (5.55)
URiM	10 637 (23.1)	11 049 (23.44)	14 565 (25.87)	11 965 (24.04)	11 575 (24.48)	11 958.2 (24.25)	11 176 (24.68)

Abbreviation: URiM, underrepresented in medicine (includes Black, Hispanic, and American Indian, Alaska Native, Native Hawaiian, and Pacific Islander students).

^a^ Race and ethnicity was defined alone or in combination with other race and ethnicities such that the total percentage will sum to greater than 100%.

^b^ The mean number, rounded to the nearest integer, and percentage of applicants from each racial and ethnic group from 2019 to 2023.

^c^ Students selected the *other* race and ethnicity category.

Representation of racial and ethnic groups among applicants also changed from 2019 to 2023 compared with 2024 (Table 1). There was a small but not significant decrease in White applicants from a mean of 26 412.8 applicants (53.57%) to 22 557 applicants (49.82%), an increase in Asian applicants from a mean of 13 931 applicants (28.25%) to 14 467 applicants (31.95%), and no significant change for Black, Hispanic, and URiM applicants (Table 1).

#### Acceptance Rates

The mean overall medical school acceptance rate in 2019 to 2023 was 39.09% compared with 44.31% in 2024, for a 5.22 percentage point (95% CI, 4.59 to 5.84 percentage points; *P* < .001) absolute increase (13.35% relative increase) (Table 2). Absolute increases in acceptance rates from the 2019 to 2023 mean vs 2024 were 40.37% to 47.47% for a difference of 7.10 percentage points (95% CI, 6.21 to 7.98 percentage points; 17.58% relative increase) for White applicants, 38.26% to 45.19% for a difference of 6.93 percentage points (95% CI, 5.78 to 8.07 percentage points; 18.11% relative increase) for Asian applicants, and 32.14% to 37.22% for a difference of 5.08 percentage points (95% CI, 2.43 to 7.72 percentage points; 15.80% relative increase) for those with other race or ethnicity (*P* < .001 for all comparisons) (Table 2). The overall URiM acceptance rate decreased significantly from 39.68% to 38.33% for an absolute difference of −1.35 percentage points (95% CI, −2.60 to −0.09 percentage points; 3.40% relative decrease; *P* = .02). There was a decrease from 36.06% to 33.08% for an absolute difference of −2.98 percentage points (95% CI, −4.74 to −1.21 percentage points; 8.26% relative decrease) in the acceptance rate for Black students (*P* < .001). There were no significant changes in acceptance rates for Hispanic or American Indian, Alaska Native, Native Hawaiian, and Pacific Islander applicants (Table 2).

**Table 2.** Acceptance Into Doctor of Medicine Programs by Race and Ethnicity

Race and ethnicity^a^	Accepted applicants, %							Absolute change (95% CI), percentage points^c^	*P* value^d^
	2019	2020	2021	2022	2023	2019-2023 mean^b^	2024		
All	39.76	39.84	34.38	39.52	41.97	39.09	44.31	5.22 (4.59 to 5.84)	<.001
American Indian, Alaska Native, Native Hawaiian, Pacific Islander	39.00	42.25	32.21	41.31	42.75	39.50	39.26	−0.24 (−5.27 to 4.79)	.92
Asian	38.59	37.57	34.25	39.50	41.41	38.26	45.19	6.93 (5.78 to 8.07)	<.001
Black	34.97	37.91	32.82	36.53	38.11	36.06	33.08	−2.98 (−4.74 to −1.21)	<.001
Hispanic	41.97	44.36	37.97	43.74	46.44	42.89	42.97	0.08 (−1.71 to 1.87)	.93
White	41.93	41.24	35.09	40.51	43.10	40.37	47.47	7.10 (6.21 to 7.98)	<.001
Other	29.97	33.26	27.95	34.19	35.35	32.14	37.22	5.08 (2.43 to 7.72)	<.001
URiM	38.82	41.33	35.47	40.38	42.43	39.68	38.33	−1.35 (−2.60 to −0.09)	.02

Abbreviation: URiM, underrepresented in medicine (includes Black, Hispanic, and American Indian, Alaska Native, Native Hawaiian, and Pacific Islander students).

^a^ Race and ethnicity was defined alone or in combination with other race and ethnicities such that the total percentage will sum to greater than 100%.

^b^ Percentage of acceptances from each racial and ethnic group from 2019 to 2023.

^c^ Absolute percentage change in acceptance rate of each racial and ethnic group for 2019 to 2023 vs 2024.

^d^ Comparison of mean acceptance rates of each racial and ethnic group in 2019 to 2023 vs the rate in 2024 using χ^2^ tests.

In 2019 to 2023, there were no differences in acceptance rates for Asian, White, or URiM applicants (eTable 1 in Supplement 1). In 2024, URiM applicants had a lower acceptance rate (38.33%) than White (47.47%; difference, −9.14 percentage points) and Asian (45.19%; difference, −6.86 percentage points) applicants (*P* < .001 for both comparisons) (eTable 1 in Supplement 1).

#### Matriculants

The number of matriculants per year increased from a mean of 18 304 matriculants in 2019 to 2023 to 19 018 matriculants in 2024, for an increase of 714 matriculants (Table 3). The number of White matriculants increased from a mean of 10 132 matriculants in 2019 to 2023 to 10 158 matriculants in 2024, for an increase of 26 matriculants, while the number of Asian matriculants increased from a mean of 5102 matriculants in 2019 to 2023 to 6288 matriculants in 2024, for an increase by 1186 matriculants. The number of URiM matriculants decreased from a mean of 4466 matriculants in 2019 to 2023 to 3963 matriculants in 2024, for a decrease of 503 URiM matriculants. Black, Hispanic, and American Indian, Alaska Native, Native Hawaiian, and Pacific Islander matriculants decreased from 1948 to 1627 matriculants (by 321 matriculants), 2438 to 2273 matriculants (by 165 matriculants), and 285 to 250 matriculants (by 35 matriculants), respectively (groups are not mutually exclusive).

**Table 3.** Race and Ethnicity of Doctor of Medicine Program Matriculants

Race and ethnicity^a^	Matriculants, No. (%)							Absolute change, No.^c^	Absolute change (95% CI), percentage points^d^	*P* value^e^
	2019	2020	2021	2022	2023	2019-2023 mean^b^	2024			
All	17 719 (100)	17 954 (100)	18 373 (100)	18 638 (100)	18 836 (100)	18 304 (100)	19 018 (100)	714	NA	NA
American Indian, Alaska Native, Native Hawaiian, Pacific Islander	286 (1.61)	299 (1.66)	267 (1.45)	272 (1.45)	301 (1.59)	285 (1.55)	250 (1.31)	−35	−0.24 (−2.25 to 1.77)	.76
Asian	4585 (25.87)	4639 (25.83)	5076 (27.62)	5500 (29.5)	5709 (30.3)	5102 (27.87)	6288 (33.06)	1186	5.19 (3.49 to 6.88)	<.001
Black	1704 (9.61)	1861 (10.36)	2251 (12.25)	1975 (10.59)	1951 (10.35)	1948 (10.64)	1627 (8.55)	−321	−2.09 (−4.01 to −0.16)	.01
Hispanic	2186 (12.33)	2399 (13.36)	2550 (13.87)	2479 (13.3)	2577 (13.68)	2438 (13.32)	2273 (11.95)	−165	−1.37 (−3.26 to 0.52)	.07
White	10 401 (58.69)	10 207 (56.85)	9857 (53.64)	10 126 (54.32)	10 071 (53.46)	10 132 (55.35)	10 158 (53.41)	26	−1.94 (−3.31 to −0.56)	.009
Other	638 (3.6)	748 (4.16)	764 (4.15)	823 (4.41)	793 (4.21)	753 (4.11)	883 (4.64)	130	0.53 (−1.45 to 2.51)	.43
URiM	4007 (22.61)	4357 (24.26)	4862 (26.46)	4505 (24.17)	4598 (24.41)	4466 (24.39)	3963 (20.83)	−503	−3.56 (−5.34 to −1.77)	<.001

Abbreviations: NA, not applicable; URiM, underrepresented in medicine (includes Black, Hispanic, and American Indian, Alaska Native, Native Hawaiian, Pacific Islander students).

^a^ Race and ethnicity was defined alone or in combination with other race and ethnicities such that the total percentage will sum to greater than 100%.

^b^ The mean number rounded to the nearest integer and percentage of matriculants from each racial and ethnic group from 2019 to 2023.

^c^ The absolute change in the number of matriculants for 2019 to 2023 vs 2024.

^d^ The absolute change in the percentage of matriculants from each race or ethnicity group for 2019 to 2023 vs 2024.

^e^ Comparison of percent matriculants from each race or ethnicity group in 2019 to 2023 vs 2024 using χ^2^ tests.

Representation of racial and ethnic groups among matriculants also shifted from 2019 to 2023 vs 2024 (Table 3). There was a small but significant decrease in White matriculants from 10 132 matriculants (55.35%) to 10 158 matriculants (53.41%), for an absolute difference of 1.94 percentage points (95% CI, −3.31 to −0.56 percentage points; 3.50% relative difference; *P* = .009), and an increase in Asian matriculants from 5102 matriculants (27.87%) to 6288 matriculants (33.06%), for an absolute difference of 5.19 percentage points (95% CI, 3.49 to 6.88 percentage points; 18.62% relative difference; *P* < .001). Overall, there was a decrease from 4466 matriculants (24.39%) to 3963 matriculants (20.83%) among URiM matriculants, for an absolute decrease of 3.56 percentage points (95% CI, −5.34 to −1.77 percentage points; 14.59% relative decrease; *P* < .001). There was a decrease from 1948 matriculants (10.64%) to 1627 matriculants (8.55%), for an absolute decrease of 2.09 percentage points (95% CI, −4.01 to −0.16 percentage points); 19.64% relative decrease) among Black matriculants (*P* = .01), and a decrease from 2438 matriculants (13.32%) to 2273 matriculants (11.95%), for an absolute difference of −1.37 percentage points (95% CI, −3.26 to 0.52 percentage points; 10.28% relative decrease), among Hispanic matriculants (*P* = .07).

### School-Level Characteristics and Change in URiM Matriculants

As shown in Table 4, from 2019 to 2023, MD programs in states with and without existing affirmative action bans had similar mean (SD) URiM representation (25.44% [11.01%] vs 27.04% [18.77%]; *P* = .56). After the 2023 SCOTUS ruling, URiM representation in institutions in states with existing affirmative action bans was unchanged (mean [SD] absolute change, 0.10 [8.11] percentage points), whereas MD programs in states without existing affirmative action bans had a decrease in mean (SD) URiM representation, from 27.04% (18.77%) to 20.89% (19.41%), for a mean (SD) 6.14 (8.93) percentage point absolute decrease (22.71% relative decrease; *P* = .001) (Table 4). While there was a decline in URiM representation after the ruling in states with and without proposed or existing anti-DEI legislation, there was no significant difference in the decline between states with and without proposed or existing anti-DEI legislation (Table 4). School ownership, mean MCAT score quartile, and geographical region were not associated with differences in school change in URiM matriculants (Table 4).

**Table 4.** School-Level Percentages of URiM Matriculants by State Policies and Institutional Characteristics

Characteristic	URiM matriculants per school, mean (SD), %		Absolute change, mean (SD), percentage points	*P* value^a^
	2019-2023	2024		
All schools (n = 151)^b^	26.69 (17.35)	21.86 (18.39)	−4.82 (9.09)	<.001
Prior statewide affirmative action ban				
No (n = 118)	27.04 (18.77)	20.89 (19.41)	−6.14 (8.93)	.001
Yes (n = 33)	25.44 (11.01)	25.33 (13.82)	0.10 (8.11)	
Statewide anti-DEI legislation				
No (n = 67)	29.96 (20.85)	25.36 (22.55)	−4.60 (8.93)	.84
Yes (n = 84)	24.98 (13.53)	19.07 (13.74)	−5.00 (9.26)	
School ownership				
Private (n = 60)	31.18 (21.60)	24.52 (22.66)	−6.65 (9.03)	.07
Public (n = 91)	23.73 (13.18)	20.10 (14.79)	−3.62 (8.97)	
Mean MCAT score, quartile				
First (lowest; n = 35)	36.57 (30.45)	31.78 (30.97)	−4.78 (9.61)	.49
Second (n = 39)	22.06 (9.92)	18.87 (12.54)	−3.19 (9.10)	
Third (n = 39)	21.87 (8.58)	16.17 (10.30)	−5.70 (9.24)	
Fourth (highest; n = 38)	27.29 (7.22)	21.65 (9.05)	−5.63 (8.54)	
Region				
Central (n = 34)	19.97 (9.74)	15.11 (10.33)	−4.86 (10.33)	.23
Northeast (n = 41)	24.98 (12.18)	18.46 (12.91)	−6.52 (8.13)	
Southern (n = 54)	3.73 (23.91)	26.79 (24.85)	−4.94 (8.33)	
Western (n = 22)	27.89 (11.46)	26.55 (13.78)	−1.33 (8.29)	

Abbreviations: DEI, diversity, equity, and inclusion; MCAT, Medical College Admissions Test; URiM, underrepresented in medicine (includes Black, Hispanic, and American Indian, Alaska Native, Native Hawaiian, and Pacific Islander students).

^a^ Comparison of mean absolute change in school percentage of URiM matriculants for 2019 to 2023 vs 2024 by state policy or institutional characteristic evaluated with *t* tests or analyses of variance.

^a^ Race and ethnicity data between 2019 and 2024 were missing for 4 schools, which were excluded from analysis.

### Sensitivity Analysis

Overall, findings were similar when considering only 2023 as the comparison period. As shown in Table 1, there was a decline in the number of applicants between 2023 and 2024 overall and in most racial and ethnic groups. As shown in eTable 2 in Supplement 1, acceptance rates for Asian and White applicants increased in 2024 compared with 2023. Acceptance rates for Black, Hispanic, and URiM applicants decreased from 2023 to 2024. In 2023, there were no differences in acceptance rates for Asian, White, or URiM applicants (eTable 3 in Supplement 1). As shown in in eTable 4 in Supplement 1, there were declines in the matriculation of Black (absolute difference, 1.80 percentage points; 16.61% relative difference; *P* < .001); Hispanic (absolute difference, 1.73 percentage points; 11.79% relative difference; *P* < .001); American Indian, Alaska Native, Native Hawaiian, and Pacific Islander (absolute difference, 0.28 percentage points; 16.90% relative difference; *P* < .001); and URiM (absolute difference 3.58 percentage points; 13.81% relative difference; *P* < .001) students in 2024 relative to 2023.

Declines in URiM matriculant representation between 2023 and 2024 were concentrated among schools located in states without prior state-level affirmative action bans (eTable 5 in Supplement 1). Proposed or existing anti-DEI legislation was not associated with differences in school change in URiM matriculants, nor were school ownership, mean MCAT score quartile, or geographical region.

## Discussion

This cross-sectional study found that in the year after the 2023 SCOTUS ruling, there was an absolute 3.56 percentage point and 14.59% relative decrease in URiM matriculants to US MD programs, from 24.39% to 20.83% relative to 2019 to 2023, reflecting an emergent disparity in acceptance rates of URiM compared with Asian and White applicants. The decline in URiM matriculation was concentrated in states without existing state-level affirmative action bans. Notably, the mean number of URiM matriculants decreased by 503 students, raising concerns about the future racial and ethnic diversity of the health care workforce.

An important finding from our study was an emergent disparity in acceptance rates of URiM applicants relative to Asian and White applicants in 2024 that was not present prior to the ban. Prior research has estimated that based on trends in medical school admissions as of 2018, it could take a century to achieve racial parity in representation among matriculants and the US poulation.^12^ The widening gap in acceptance rates to the detriment of URiM applicants after the 2023 SCOTUS ruling could prolong this timeline and raises doubts about its feasibility.

The 2023 SCOTUS decision was grounded in the principle of meritocracy. However, the concept of meritocracy in medical education exists within a broader context of persistent systemic racial and ethnic discrimination, including unequal access to educational opportunities.^13,14,15,16^ While the ruling was intended to increase fairness by making admissions decisions race neutral, the emergence of disparity in acceptances rate between URiM and White students may suggest that the ruling reflects the continued influence of longstanding structural barriers.

It is important to contextualize the decrease by 503 URiM medical school matriculants in 2024. This figure is nearly equivalent to the annual number of URiM matriculants to all historically Black colleges and universities (HBCUs) and Puerto Rico Hispanic-serving institutions and nearly double the projected loss in 2019 of 355 Black graduates resulting from the closure of HBCUs after the 1910 Flexner Report.^17^

Our study also found that declines in URiM representation occurred uniformly across nearly all medical school characteristics assessed. Only the existence of a prior state-level ban on affirmative action was associated with changes in URiM matriculants after the SCOTUS ruling, with declines in URiM representation occurring principally within medical schools located in states without a prior, state-level affirmative action ban. This finding is not unexpected given that we would not anticipate a federal affirmative action ban to influence medical schools in states with affirmative action bans already in place. The concentration of the decline of URiM matriculants in states without prior affirmative action bans 1 year after the 2023 SCOTUS decision suggests that this decline may be an outcome associated with the decision itself rather than other factors, such as the COVID-19 pandemic or changes in applicant qualifications.

Prior literature has suggested that state-level affirmative action bans had the greatest effect on the most selective schools, with URiM students redistributing to less selective schools.^18^ However, URiM students experienced similar declines after the SCOTUS ruling in aggregate at the most and least selective schools as measured by mean matriculant standardized exam scores. These findings suggest that a redistribution of URiM students did not occur but instead there was an overall decrease in URiM students from MD-granting institutions after the SCOTUS ruling.

It is important to note the potential implications of a less inclusive physician workforce. The US faces a public health crisis and a projected nationwide shortage of more than 130 000 physicians by 2030 that will disproportionately impact medically underserved areas.^19,20^ URiM physicians are more likely to provide medical care to uninsured and Medicaid patients and individuals living in medically underserved communities.^21,22^ A decline in the number of URiM physicians has significant implications for the future of health care access, especially for patients with the greatest need.^22^

Furthermore, a decline in physician workforce inclusivity has implications for scientific progress. Diversity in medical research has been associated with greater creativity, improved problem solving, and advancements that benefit the broader society.^23,24,25^ Racial and ethnic diversity of scientific teams is strongly correlated with scientific impact.^23^ Consequently, a reduction in the representation of URiM physicians may compromise scientific discovery and limit the quality of science the biomedical workforce can deliver.

Lastly, it is important to note that a diverse learning environment benefits all learners. A study of 7000 White medical students demonstrated that those in racially diverse learning environments felt better equipped to care for patients from racial and ethnic backgrounds different from their own compared with White medical students in less racially and ethnically diverse environments.^26^ Another study of more than 3000 Asian, Hispanic, and White students demonstrated that positive social interactions with Black faculty were associated with lower implicit bias.^27^ These studies highlight the critical role that racial and ethnic diversity plays in the professional development of physicians.^28^

### Limitations

Our study has several limitations. While to our knowledge, it is the first comprehensive, national study of the racial and ethnic landscape of medical school applications, admissions, and matriculation after the 2023 SCOTUS ruling, we were limited to 1 year of postruling data. Implementation of pathway programs and changes in admission policies may require time to yield results and could stabilize the decline in URiM acceptances and matriculation, although implementing such programs may be challenging amid current state and federal efforts to dismantle DEI initiatives. Furthermore, due to the observational nature of this study, we cannot rule out the possibility that other factors may have influenced URiM acceptance rates and representation among matriculants; these factors may include applicant characteristics (such as first-time vs repeat applicants, standardized test scores, and grade point average), year-to-year changes in the number of applicants and characteristics of MD programs to which students apply, and the surge in applicants due to the COVID-19 pandemic. For instance, we observed an increase in URiM applicants in 2021, which coincided with the social justice movement after the murder of George Floyd. However, it is important to note that the decline in URiM representation reported in this study was concentrated in states without preexisting affirmative action bans, suggesting that the observed national decline was more likely attributable to the 2023 SCOTUS ruling than residual effects of the COVID-19 pandemic or changes in applicant characteristics. While our analyses were restricted to race and ethnicity, it is possible that the SCOTUS ruling affected representation of other groups based on characteristics such as disability status. Additionally, while we observed increases in Asian representation after the 2023 SCOTUS ruling, this trend should be interpreted with nuance given that Asian individuals remain underrepresented in leadership roles within medicine and academia, a dynamic often described as the “bamboo ceiling.”^29^ Additionally, Asian medical students face significant structural barriers in medical education, including lower likelihood of induction into medical honor societies and placement into residency programs.^13,30,31,32^ Finally, acceptance and matriculation patterns among subgroups within the Asian applicant pool are likely heterogeneous, necessitating the future disaggregation of Asian subgroups to capture the diversity of admissions and matriculation experiences within this population.^33^

## Conclusions

In this cross-sectional study, we observed a 3.56 percentage point absolute and 14.59% relative decrease in URiM matriculants to US MD programs, as well as an emergent disparity in acceptance rates of URiM compared with Asian and White applicants after the 2023 SCOTUS ruling on race-conscious admissions policies. The decline in URiM representation may have significant and long-lasting implications for racial and ethnic diversity in the physician workforce.

## Notes

Supplement 1. **eTable 1.** Comparisons of Acceptance Rates Among URiM Applicants and Asian or White Applicants in 2019-2023 and 2024 **eTable 2.** Rate of Acceptance Into MD Programs According to Race and Ethnicity in 2023 and 2024 **eTable 3.** Comparisons of Acceptance Rates Among URiM Applicants and Asian or White Applicants in 2023 and 2024 **eTable 4.** Representation of Race and Ethnicity Groups Among MD Program Matriculants, 2023-2024 **eTable 5.** Comparison of School-Level Percentages of URiM Matriculants by State Policies and Institutional Characteristics, 2023 and 2024

Supplement 2.
Data Sharing Statement

## References

[1] Cohen JJ. The consequences of premature abandonment of affirmative action in medical school admissions. JAMA. 2003;289(9):1143-1149. doi:10.1001/jama.289.9.1143 12622585

[2] Holzer H, Neumark D. Assessing Affirmative Action. J Econ Lit. 2000;38(3):483-568. doi:10.1257/jel.38.3.483

[3] Hurley L. Supreme Court strikes down college affirmative action programs. NBC News. 2023. Accessed July 11, 2025. https://www.nbcnews.com/politics/supreme-court/supreme-court-strikes-affirmative-action-programs-harvard-unc-rcna66770

[4] Sherman M. Divided Supreme Court outlaws affirmative action in college admissions, says race can’t be used. Associated Press. Accessed July 11, 2025. https://apnews.com/article/supreme-court-affirmative-action-college-race-f83d6318017ec9b9029b12ee2256e744

[5] Totenberg N. Supreme Court guts affirmative action, effectively ending race-conscious admissions. National Public Radio. Accessed July 11, 2025. https://www.npr.org/2023/06/29/1181138066/affirmative-action-supreme-court-decision#:~:text=In%20a%20historic%20decision%2C%20the,and%20universities%20across%20the%20country2023

[6] Ly DP, Essien UR, Olenski AR, Jena AB. Affirmative action bans and enrollment of students from underrepresented racial and ethnic groups in U.S. public medical schools. Ann Intern Med. 2022;175(6):873-878. doi:10.7326/M21-4312 35500257

[7] Association of American Medical Colleges. New AAMC data on medical school applicants and enrollment in 2024. Association of American Medical Colleges. 2025. Accessed July 11, 2025. https://www.aamc.org/news/press-releases/new-aamc-data-medical-school-applicants-and-enrollment-2024

[8] Nguyen M, Chaudhry SI, Asabor E, . Variation in research experiences and publications during medical school by sex and race and ethnicity. JAMA Netw Open. 2022;5(10):e2238520. doi:10.1001/jamanetworkopen.2022.38520 36282497 PMC9597391

[9] Nguyen M, Chaudhry SI, Desai MM, . Rates of medical student placement into graduate medical education by sex, race and ethnicity, and socioeconomic status, 2018-2021. JAMA Netw Open. 2022;5(8):e2229243. doi:10.1001/jamanetworkopen.2022.29243 36018592 PMC9419015

[10] Nguyen M, Chaudhry SI, Desai MM, . Association of sociodemographic characteristics with US medical student attrition. JAMA Intern Med. 2022;182(9):917-924. doi:10.1001/jamainternmed.2022.2194 35816334 PMC9274446

[11] The Chronicle of Higher Education. DEI legislation tracker: explore where college diversity, equity, and inclusion efforts are under attack. The Chronicle of Higher Education. Updated August 30, 2024. Accessed July 11, 2025. https://www.chronicle.com/article/here-are-the-states-where-lawmakers-are-seeking-to-ban-colleges-dei-efforts

[12] Bennett CL, Yiadom MYAB, Baker O, Marsh RH. Examining parity among Black and Hispanic resident physicians. J Gen Intern Med. 2021;36(6):1722-1725. doi:10.1007/s11606-021-06650-7 33629264 PMC8175607

[13] Hill KA, Desai MM, Chaudhry SI, . Association of Marginalized Identities With Alpha Omega Alpha Honor Society and Gold Humanism Honor Society membership among medical students. JAMA Netw Open. 2022;5(9):e2229062. doi:10.1001/jamanetworkopen.2022.29062 36069984 PMC9453541

[14] Hill KA, Samuels EA, Gross CP, . Assessment of the prevalence of medical student mistreatment by sex, race/ethnicity, and sexual orientation. JAMA Intern Med. 2020;180(5):653-665. doi:10.1001/jamainternmed.2020.0030 32091540 PMC7042809

[15] Nguyen M, Chaudhry SI, Desai MM, . Association of mistreatment and discrimination with medical school attrition. JAMA Pediatr. 2022;176(9):935-937. doi:10.1001/jamapediatrics.2022.1637 35639402 PMC9157380

[16] Teshome BG, Desai MM, Gross CP, . Marginalized identities, mistreatment, discrimination, and burnout among US medical students: cross sectional survey and retrospective cohort study. BMJ. 2022;376:e065984. doi:10.1136/bmj-2021-065984 35318190 PMC8938931

[17] Campbell KM, Corral I, Infante Linares JL, Tumin D. Projected estimates of African American medical graduates of closed historically Black medical schools. JAMA Netw Open. 2020;3(8):e2015220. doi:10.1001/jamanetworkopen.2020.15220 32816033 PMC7441360

[18] Garces LM, Mickey-Pabello D. Racial diversity in the medical profession: the impact of affirmative action bans on underrepresented student of color matriculation in medical schools. J Higher Educ. 2015;86(2):264-294. doi:10.1080/00221546.2015.11777364 26052161 PMC4454423

[19] Saha S. Taking diversity seriously: the merits of increasing minority representation in medicine. JAMA Intern Med. 2014;174(2):291-292. doi:10.1001/jamainternmed.2013.12736 24378744

[20] Zhang X, Lin D, Pforsich H, Lin VW. Physician workforce in the United States of America: forecasting nationwide shortages. Hum Resour Health. 2020;18(1):8. doi:10.1186/s12960-020-0448-3 32029001 PMC7006215

[21] Marrast LM, Zallman L, Woolhandler S, Bor DH, McCormick D. Minority physicians’ role in the care of underserved patients: diversifying the physician workforce may be key in addressing health disparities. JAMA Intern Med. 2014;174(2):289-291. doi:10.1001/jamainternmed.2013.12756 24378807

[22] Snyder JE, Upton RD, Hassett TC, Lee H, Nouri Z, Dill M. Black representation in the primary care physician workforce and its association with population life expectancy and mortality rates in the US. JAMA Netw Open. 2023;6(4):e236687. doi:10.1001/jamanetworkopen.2023.6687 37058307 PMC10105312

[23] AlShebli BK, Rahwan T, Woon WL. The preeminence of ethnic diversity in scientific collaboration. Nat Commun. 2018;9(1):5163. doi:10.1038/s41467-018-07634-8 30514841 PMC6279741

[24] Hofstra B, Kulkarni VV, Munoz-Najar Galvez S, He B, Jurafsky D, McFarland DA. The diversity-innovation paradox in science. Proc Natl Acad Sci U S A. 2020;117(17):9284-9291. doi:10.1073/pnas.1915378117 32291335 PMC7196824

[25] Hong L, Page SE. Groups of diverse problem solvers can outperform groups of high-ability problem solvers. Proc Natl Acad Sci U S A. 2004;101(46):16385-16389. doi:10.1073/pnas.0403723101 15534225 PMC528939

[26] Saha S, Guiton G, Wimmers PF, Wilkerson L. Student body racial and ethnic composition and diversity-related outcomes in US medical schools. JAMA. 2008;300(10):1135-1145. doi:10.1001/jama.300.10.1135 18780842

[27] van Ryn M, Hardeman R, Phelan SM, . Medical school experiences associated with change in implicit racial bias among 3547 students: a medical student CHANGES study report. J Gen Intern Med. 2015;30(12):1748-1756. doi:10.1007/s11606-015-3447-7 26129779 PMC4636581

[28] Morrison E, Grbic D. Dimensions of diversity and perception of having learned from individuals from different backgrounds: the particular importance of racial diversity. Acad Med. 2015;90(7):937-945. doi:10.1097/ACM.0000000000000675 25719675

[29] Morris DB, Gruppuso PA, McGee HA, Murillo AL, Grover A, Adashi EY. Diversity of the national medical student body—four decades of inequities. N Engl J Med. 2021;384(17):1661-1668. doi:10.1056/NEJMsr2028487 33913645

[30] Nguyen M, Chaudhry SI, Desai MM, . Rates of medical student placement into graduate medical education by sex, race and ethnicity, and socioeconomic status, 2018-2021. JAMA Netw Open. 2022;5(8):e2229243. doi:10.1001/jamanetworkopen.2022.29243 36018592 PMC9419015

[31] Nguyen M, Mason HRC, Russell R, . Leave of absence and medical student placement into graduate medical education by race and ethnicity. JAMA. 2024;331(18):1588-1590. doi:10.1001/jama.2024.4797 38619837 PMC11019442

[32] Boatright D, Ross D, O’Connor P, Moore E, Nunez-Smith M. Racial disparities in medical student membership in the Alpha Omega Alpha Honor Society. JAMA Intern Med. 2017;177(5):659-665. doi:10.1001/jamainternmed.2016.9623 28264091 PMC5818775

[33] Nguyen M, Boatright D, Fancher TL. Disaggregating Asian American data to enhance medical education and patient care. JAMA Netw Open. 2024;7(11):e2444485. doi:10.1001/jamanetworkopen.2024.44485 39560948

