# Supplementary material for: Medical School Admissions After the Supreme Court’s 2023 Affirmative Action Ruling
Source: JAMA Netw Open. 2025 Aug 26;8(8):e2527008. doi: 10.1001/jamanetworkopen.2025.27008 (PMC12381671; doi:10.1001/jamanetworkopen.2025.27008)
Supplement: Supplement 1. — eTable 1. Comparisons of Acceptance Rates Among URiM Applicants and Asian or White Applicants in 2019-2023 and 2024 eTable 2. Rate of Acceptance Into MD Programs According to Race and Ethnicity in 2023 and 2024 eTable 3. Comparisons of Acceptance Rates Among URiM Applicants and Asian or White Applicants in 2023 and 2024 eTable 4. Representation of Race and Ethnicity Groups Among MD Program Matriculants, 2023-2024 eTable 5. Comparison of School-Level Percentages of URiM Matriculants by State Policies and Institutional Characteristics, 2023 and 2024 [file jamanetwopen-e2527008-s001.pdf]

Supplemental Online Content

Nguyen M, Hajduk AM, Fancher TL, et al. Medical school admissions after the Supreme Court's 2023 affirmative action ruling. *JAMA Netw Open*. 2025;8(8):e2527008. doi:10.1001/jamanetworkopen.2025.27008

eTable 1. Comparisons of Acceptance Rates Among URiM Applicants and Asian or White Applicants in 2019-2023 and 2024

eTable 2. Rate of Acceptance Into MD Programs According to Race and Ethnicity in 2023 and 2024

eTable 3. Comparisons of Acceptance Rates Among URiM Applicants and Asian or White Applicants in 2023 and 2024

eTable 4. Representation of Race and Ethnicity Groups Among MD Program Matriculants, 2023-2024

eTable 5. Comparison of School-Level Percentages of URiM Matriculants by State Policies and Institutional Characteristics, 2023 and 2024

This supplemental material has been provided by the authors to give readers additional information about their work.

© 2025 Nguyen M et al. *JAMA Network Open*.

eTable 1. Comparisons of Acceptance Rates among URiM Applicants and Asian or White Applicants in 2019-23 and 2024

Comparison groups and year(s)	URiM acceptance rate (%)	Asian or White acceptance rate (%)	Absolute % Difference[1] (p-value[2])
URiM and Asian, 2019-23	39.68	38.26 (Asian)	+1.42 (0.07)
URiM and White, 2019-23		40.37 (White)	-0.69 (0.76)
URiM and Asian, 2024	38.33	45.19 (Asian)	-6.86 (p<0.001)
URiM and White, 2024		47.47 (White)	-9.14 (p<0.001)

[1]Absolute percent difference in acceptance rates between race or ethnicity groups in specified year(s)

[2]Comparison of percent difference in acceptance rates between URiM and White or Asian groups in specified year(s)

AIAN/NHPI: American Indian, Alaska Native, Native Hawaiian, Pacific Islander.

URiM: underrepresented in medicine includes Black, Hispanic, and AIAN/NHPI students.

© 2025 Nguyen M et al. *JAMA Network Open*.

eTable 2. Rate of Acceptance into MD Programs According to Race and Ethnicity in 2023 and 2024

Acceptances, %[1]	2023	2024	Absolute % change[2] (p-value)[3]
All students	41.97	44.31	+2.34 (<0.001)
AIAN/NHPI	42.75	39.26	-3.49 (0.18)
Asian	41.41	45.19	+3.78 (<0.001)
Black	38.11	33.08	-5.03 (<0.001)
Hispanic	46.44	42.97	-3.47 (<0.001)
Other race/ethnicity	35.35	37.22	+1.87 (0.18)
URiM	42.43	38.33	-4.10 (<0.001)
White	43.10	47.47	+4.37 (<0.001)

[1]Race and ethnicity is defined as alone or in combination with other race and ethnicities such that the total percentage will add up to greater than 100%.

[2]Absolute percent change in acceptance rate or each race or ethnicity group between 2023 and 2024

[3]Comparison of average acceptance rates from 2023 and 2024 using chi-square tests

AIAN/NHPI: American Indian, Alaska Native, Native Hawaiian, Pacific Islander.

URiM: underrepresented in medicine includes Black, Hispanic, and AIAN/NHPI students.

© 2025 Nguyen M et al. *JAMA Network Open*.

eTable 3. Comparisons of Acceptance Rates among URiM Applicants and Asian or White Applicants in 2023 and 2024

Comparison groups and year(s)	URiM acceptance rate (%)	Asian or White acceptance rate (%)	Absolute % Difference[1] (p-value[2])
URiM and Asian, 2023	42.43	41.41 (Asian)	+1.02 (0.10)
URiM and White, 2023		43.10 (White)	-0.67 (0.23)
URiM and Asian, 2024	38.33	45.19 (Asian)	-6.86 (p<0.001)
URiM and White, 2024		47.47 (White)	-9.14 (p<0.001)

[1]Absolute percent difference in acceptance rates between URiM and White or Asian group in specified year(s)

[2]Comparison of percent difference in acceptance rates between URiM and White or Asian group in specified year(s)

AIAN/NHPI: American Indian, Alaska Native, Native Hawaiian, Pacific Islander.

URiM: underrepresented in medicine includes Black, Hispanic, and AIAN/NHPI students.

© 2025 Nguyen M et al. *JAMA Network Open*.

eTable 4. Representation of Race and Ethnicity Groups among MD Program Matriculants, 2023-24

Matriculants, n(%)[1]	2023	2024	Absolute change,[2] n	Absolute % change[3] (p-value[4])
All students	18,836 (100)	19,018 (100)	+182	--
AIAN/NHPI	301 (1.59)	250 (1.31)	-51	-0.28 (<0.001)
Asian	5709 (30.3)	6288 (33.06)	+579	+2.76 (<0.001)
Black	1951 (10.35)	1627 (8.55)	-324	-1.80 (<0.001)
Hispanic	2577 (13.68)	2273 (11.95)	-304	-1.73 (<0.001)
Other race/ethnicity	793 (4.21)	883 (4.64)	+90	+0.43 (0.001)
URiM	4598 (24.41)	3963 (20.83)	-635	-3.58 (<0.001)
White	10071 (53.46)	10158 (53.41)	+87	-0.05 (0.11)

[1]Race and ethnicity is defined as alone or in combination with other race and ethnicities such that the total percentages will add up to greater than 100%.

[2]Absolute change in number of matriculants between 2023 and 2024.

[3]Absolute change in percent of matriculants from each race or ethnicity group between 2023 and 2024.

[4]Comparison of percent matriculants from each race or ethnicity group in 2023 and 2024 using chi-square tests.

AIAN/NHPI: American Indian, Alaska Native, Native Hawaiian, Pacific Islander.

URiM: underrepresented in medicine includes Black, Hispanic, and AIAN/NHPI students.

© 2025 Nguyen M et al. *JAMA Network Open*.

eTable 5. Comparison of School-level Percentages of URiM Matriculants by State Policies and Institutional Characteristics, 2023 and 2024

	Mean percentage of URiM matriculants per school, % (SD)		Mean percent absolute change (SD)	*p*-value[2]
	2023	2024		
All schools, N=155[1]	25.73 (17.77)	22.74 (19.41)	-2.99 (8.76)	**<0.001**
Prior statewide affirmative action ban				
No (n=120)	25.35 (18.42)	21.20 (19.52)	-4.15 (8.36)	**0.004**
Yes (n=35)	27.04 (15.47)	28.02 (18.36)	+0.98 (9.05)	
State-wide anti-DEI legislation				
No (n=69)	29.31 (21.49)	26.72 (23.96)	-2.58 (8.34)	0.72
Yes (n=86)	22.86 (13.55)	19.54 (14.15)	-3.32 (9.11)	
School ownership				
Private (n=62)	30.63 (22.06)	26.07 (24.27)	-4.56 (9.11)	0.12
Public (n=93)	22.46 (13.37)	20.52 (15.10)	-1.95 (8.40)	
Average MCAT, quartile				
1st (lowest; n=38)	35.12 (30.59)	33.88 (31.77)	-1.24 (8.96)	0.20

© 2025 Nguyen M et al. *JAMA Network Open*.

eTable 5 (continued). Comparison of School-level Percentages of URiM Matriculants by State Policies and Institutional Characteristics, 2023 and 2024

2nd (n=39)	20.94 (10.01)	18.87 (12.54)	-2.08 (8.77)	
3rd (n=39)	20.20 (7.27)	16.17 (10.30)	-4.03 (9.11)	
4th (highest; n=39)	26.90 (8.39)	22.32 (9.87)	-4.57 (8.08)	
Region				
Central (n=34)	18.19 (8.44)	15.11 (10.33)	-3.08 (9.03)	0.27
Northeast (n=41)	22.91 (12.72)	18.46 (12.91)	-4.45 (8.16)	
Southern (n=56)	30.60 (23.02)	27.23 (24.71)	-3.37 (9.18)	
Western (n=24)	29.87 (16.99)	30.37 (19.80)	0.49 (7.91)	

[1]Two schools missing race/ethnicity data between 2021-24 excluded from analysis

[2]Comparison of mean absolute change in schools' percent URiM matriculants between 2023 and 2024 according to state policy or institutional characteristic, evaluated with t-tests or analyses of variance

URiM: underrepresented in medicine includes Black, Hispanic, and AIAN/NHPI students.

© 2025 Nguyen M et al. *JAMA Network Open*.
