# Supplementary material for: Medical School Admissions After the Supreme Court’s 2023 Affirmative Action Ruling
Source: JAMA Netw Open. 2025 Aug 26;8(8):e2527008. doi: 10.1001/jamanetworkopen.2025.27008 (PMC12381671; doi:10.1001/jamanetworkopen.2025.27008)
Supplement: Supplement 2. — Data Sharing Statement [file jamanetwopen-e2527008-s002.pdf]

Data Sharing Statement

Nguyen. Medical School Admissions After the Supreme Court's 2023 Affirmative Action Ruling. *JAMA Netw Open*. Published online August 26, 2025. doi:10.1001/jamanetworkopen.2025.27008

Data
Data available: No
